# Application of Digital Medicine to the Diagnosis and Treatment of Head and Neck Tumors

**DOI:** 10.1002/cnr2.70556

**Published:** 2026-04-22

**Authors:** Bin He, Yanghao Hu, Zhikai Wang, Yejingwen Tong, Shengqi Gan, Yaojie Zhu, Chongyang Wang, Dong Ye

**Affiliations:** ^1^ Department of Otorhinolaryngology‐Head and Neck Surgery, the Affiliated Lihuili Hospital Ningbo University Ningbo Zhejiang China; ^2^ Department of Otorhinolaryngology Daqi Community Health Service Center Ningbo Zhejiang China; ^3^ Department of Otorhinolaryngology Lingshan Community Health Center Ningbo Zhejiang China

**Keywords:** digital medicine, early diagnosis, head and neck tumors, precision treatment, prognosis assessment

## Abstract

**Background:**

The annual incidence of head and neck tumors (HNTs) is increasing, and their complex anatomical structure makes diagnosis, treatment, and prognosis assessment highly challenging. This narrative review aims to summarize the application status of digital medicine in the diagnosis, treatment, and prognosis evaluation of HNTs.

**Recent Findings:**

Digital medical technologies have advanced rapidly in recent years. Digital imaging (CT, MRI, and ultrasonography) is widely used for tumor localization, progression monitoring, and treatment efficacy evaluation. Artificial intelligence optimizes tumor detection and radiotherapy planning via deep learning models, improving diagnostic accuracy and therapeutic effects. 3D printing shows significant clinical value in preoperative planning and personalized surgical navigation. Current research limitations are also identified.

**Conclusion:**

Digital medicine is expected to further promote the early diagnosis of HNTs, support the establishment of personalized precision treatment strategies, and ultimately improve the quality of life and prognosis of patients.

**Abbreviations**
ADC: apparent diffusion coefficient
AI: artificial intelligence
APCs: antigen‐presenting cells
AR: augmented reality
AUC: area under the curve
AUROC: area under the receiver operating characteristic curve
BLT: benign laryngeal tumors
CAD: computer‐aided diagnosis
CCRT: concurrent chemoradiotherapy
CD28: cluster of differentiation 28
CD80: cluster of differentiation 80
CDUS: color Doppler ultrasound
CECT: contrast‐enhanced CT
ceMRI: contrast‐enhanced magnetic resonance imaging
CNN: convolutional neural network
CT: computed tomography
DCE‐MRI: dynamic contrast enhanced magnetic resonance imaging
DCs: dendritic cells
DFS: disease‐free survival
DL: deep learning
DMFS: distant metastasis‐free survival
DW‐MRI: diffusion‐weighted magnetic resonance imaging
EBV: epstein–barr virus
EGFR: epidermal growth factor receptor
ETE: extrathyroidal extension
EVs: extracellular vesicles
HNSCC: head and neck squamous cell carcinoma
HNTs: head and neck tumors
HPV: human papillomavirus
IAUC: incremental area under the curve
ICT: induction chemotherapy
IFN‐γ: interferon—γ
IMRT: intensity‐modulated radiotherapy
LA‐NPC: locoregionally advanced nasopharyngeal carcinoma
LCA: laryngeal cancer
LN: lymph nodes
MHC: major histocompatibility complex
ML: machine learning
MRI: magnetic resonance imaging
MRS: magnetic resonance spectroscopy
NPC: nasopharyngeal carcinoma
OPSCC: oropharyngeal squamous cell carcinoma
OS: overall survival
OTC: organotypic culture
PD‐1: programmed cell death protein 1
PD‐L1: programmed death‐ligand 1
PDO: patient derived organoids
PFS: progression‐free survival
PPSI: parapharyngeal space involvement
PRELCA: precancerous laryngeal lesions
RT: radiation therapy
SRS: stimulated Raman scattering
TCR: T cell receptor
TLG‐T: tesion glycolysis—tumor
TSCC: tongue squamous cell carcinoma
VR: virtual reality

## 1 Introduction

According to the Global Cancer Statistics of 2020, HNC ranked as the third most prevalent cancer worldwide, with 1 464 550 new cases and 487 993 deaths. This accounted for 7.6% of all cancers and 4.8% of all cancer‐related deaths [1]. The most common type is head and neck squamous cell carcinoma (HNSCC), accounting for more than 90%. The incidence rate in men is higher than that in women, and nasopharyngeal carcinoma (NPC) accounts for approximately 40% of HNTs in endemic regions (e.g., Southeast Asia, Southern China) [2], while oral cavity and oropharyngeal carcinoma are the most common subtypes in global non‐endemic regions. The main risk factors include tobacco smoking, alcohol consumption, human papillomavirus (HPV) infection, Epstein–Barr virus infection, and genetic factors. The 5‐year survival rate is 60%–90% at the early‐stage (stages I–II) and 30%–50% at the late stage (stages III–IV). Due to the complexity of the anatomical location of HNTs, early diagnosis, treatment, and prognostic assessment are challenging.

At present, the diagnosis of HNTs is mainly based on clinical symptoms (such as long‐term hoarseness, difficulty swallowing, recurrent nosebleeds, and earache), imaging (such as CT, MRI, positron emission tomography (PET)/(CT), electronic nasopharyngoscopy, dynamic laryngoscopy, and video nasal endoscopy), and biopsy with pathological examination.

The treatment of HNTs is mainly divided into surgical and non‐surgical treatments. Currently, minimally invasive resection of the lesion and selective cervical lymph node dissection (grade I–IV) or scapulohyoid cervical lymph node dissection (grade I–III) are widely performed to remove cervical lymph nodes while preserving non‐lymph node tissue. Presently, widely used non‐surgical treatments include molecular biology‐ and genomics‐based treatments, targeted therapy, and immunotherapy. Immunotherapy is an important non‐surgical treatment for HNTs, and digital medicine technologies such as artificial intelligence have been gradually applied to predict the response of HNT patients to PD‐1/PD‐L1 immunotherapy and screen potential beneficiaries [3, 4, 5].

With the continuous progress of scientific research, in addition to the conventional diagnostic methods mentioned above, breath analysis, exosomes, and extracellular vesicles (EVs) have been explored for their potential use in diagnosing HNTs. Salivary protein biomarkers and serum plasma have also been evaluated for use in diagnosing HNTs [6]. Regarding treatment, in molecular biology and genomics, the difference in molecular characteristics between HPV‐positive and HPV‐negative HNSCC is an important research direction [7]. HPV‐positive tumors have a relatively good prognosis [8], and digital gene expression analysis technology has been applied to the molecular diagnosis of thyroid tumors [9]. Furthermore, immune checkpoint inhibitors have demonstrated good clinical efficacy in the treatment of HNTs, offering a new therapeutic option for patients with refractory HNTs. However, clinical practice of HNTs still faces challenges such as difficult early diagnosis, inaccurate tumor staging and lack of individualized treatment plans [10, 11, 12, 13, 14, 15], and digital medicine technologies provide new solutions for solving these problems, such as AI‐based biomarker screening and radiomics‐based tumor heterogeneity analysis.

**FIGURE 1:**
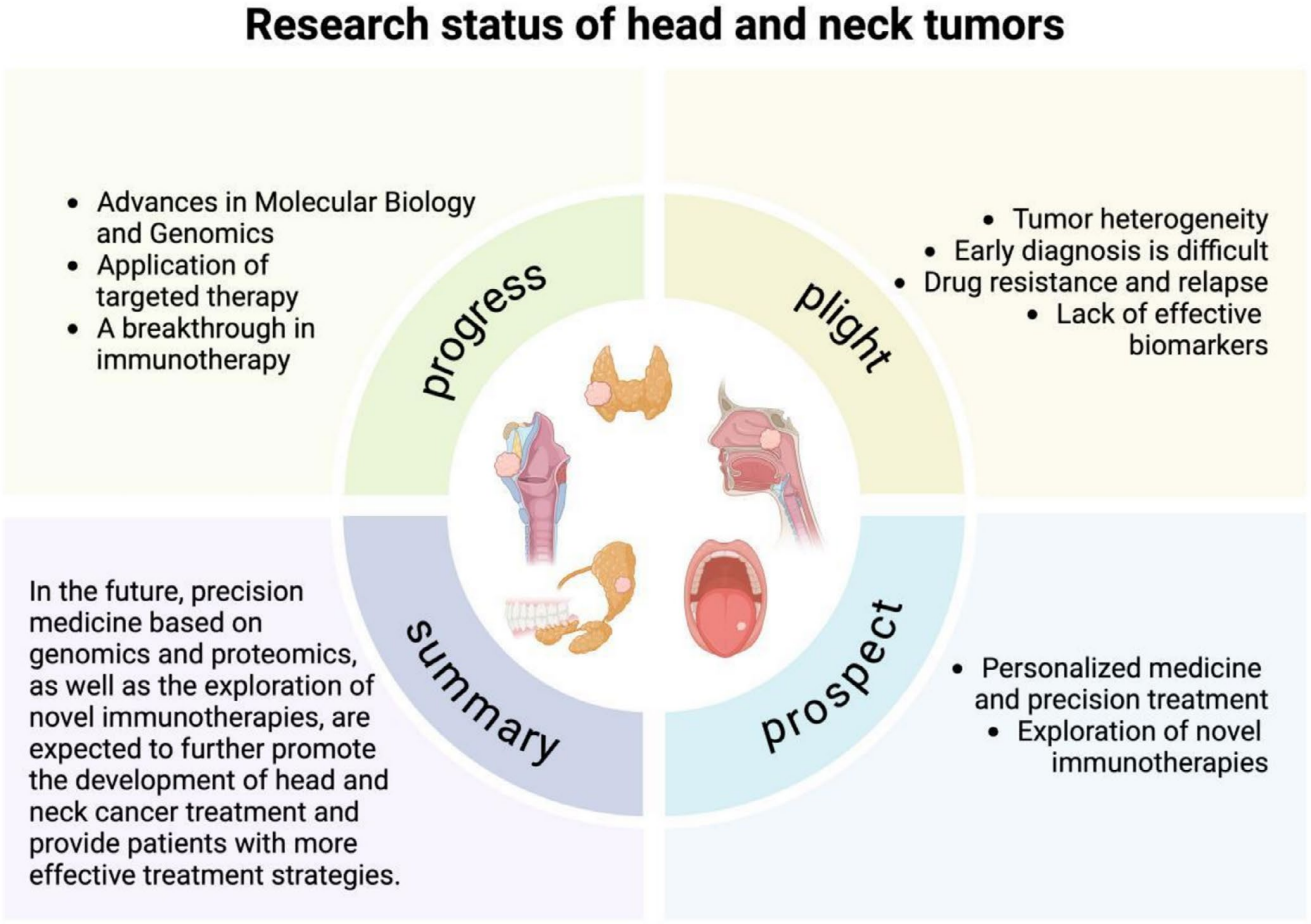
This illustration presents the research status of head and neck tumors, covering progress (advances in molecular biology/genomics, targeted therapy, immunotherapy breakthroughs), plight (tumor heterogeneity, late diagnosis, drug resistance/relapse, lack of biomarkers), prospect (personalized/precision treatment, novel immunotherapies exploration), and summary (precision medicine and novel immunotherapies to improve treatment).

The rapid development of digital medicine, particularly in the fields of imaging technology, artificial intelligence, and 3D technology, has provided new directions and means for diagnosing, treating, and assessing the prognosis of HNTs [16]. Digital imaging technologies, such as CT, MRI, and ultrasonography, have become indispensable tools in the diagnosis and treatment of head and neck tumors, enabling doctors to locate tumors more accurately, guide personalized treatment plans, and monitor treatment efficacy in real‐time [17]. The introduction of artificial intelligence, especially in the analysis and in‐depth study of radiomics, has made the detection of early‐stage tumors and the development of individualized treatment plans more reliable and effective. Additionally, the application of 3D technology (including 3D imaging, 3D printing, and 3D organoid culture) to preoperative planning and simulated surgery has gradually demonstrated its advantages in treating complex tumors. The purpose of this review is to summarize the latest progress of digital medicine in the diagnosis and treatment of HNTs, including the status of the application of digital imaging, artificial intelligence (AI), and 3D technologies to its diagnosis, treatment, and prognosis assessment, and to discuss and analyze their development trends and future research directions. And to discuss and analyze their development trends and future research directions (Figure 1).

## 2 Methodology

This is a narrative review focusing on the application of digital medicine in HNTs. The literature search was conducted in PubMed, Web of Science, CNKI and Wanfang Database from January 2019 to December 2025 using the key words including “head and neck tumors”, “digital medicine”, “artificial intelligence”, “3D printing”, “digital imaging”, “early diagnosis”, “precision treatment” and “prognosis assessment”. The inclusion criteria were: (1) original clinical and basic research studies on the application of digital medical technology in HNTs; (2) review articles summarizing the progress of digital medicine in oncology; (3) studies with clear experimental design and available clinical data. The exclusion criteria were: (1) studies not related to digital medicine; (2) case reports with small sample size and no statistical significance; (3) non‐English and non‐Chinese literatures with no available translations. After literature screening, a total of 95 relevant studies were included for synthesis and analysis, with a focus on the latest research progress of digital imaging, artificial intelligence and 3D technology in HNTs.

## 3 Research Status of Digital Medicine in the Diagnosis and Treatment of Head and Neck Tumors

### 3.1 Digital Imaging Advances in HNTs

Digital imaging is widely used in the medical field, playing a key role in disease diagnosis, treatment, and prognosis evaluation. MRI, CT, and B‐ultrasound are widely used in evaluating HNTs. Here, we discuss the research progress in digital imaging for the diagnosis, treatment, and prognosis evaluation of HNTs over the past 5 years.

#### 3.1.1 Application of Digital Imaging to the Diagnosis of Head and Neck Tumors

Multiple images can improve diagnostic accuracy in complementary ways. Terzidis et al. [18] used a modern hybrid imaging approach to accurately determine the boundary of 5 mm tumors. King et al. used MRI and endoscopy to diagnose early nasopharyngeal carcinoma (NPC): 2.9% of participants with stage I NPC were diagnosed by endoscopy, and 17.6% of participants with NPC (stage I/4, stage II/1, and stage III/1) were diagnosed by MRI. MRI played a complementary role in the endoscopic diagnosis of NPC and could help diagnose early‐stage endoscopic recessive NPC. Additionally, the sensitivity, specificity, and accuracy of endoscopy were 76.5%, 97.5%, and 94.9%, respectively, and the sensitivity, specificity, and accuracy of MRI were 91.2%, 97.5%, and 96.7%, respectively. MRI has a higher diagnostic accuracy for NPC than endoscopy [19]. Wong et al. utilized a convolutional neural network (CNN) scoring system ranging from 0 to 1 in T2‐weighted MRI and determined that the threshold for distinguishing NPCs confined to the nasopharynx from benign hyperplasia was greater than 0.71, enabling CNNs to detect early NPCs with a high sensitivity of 92.4% and a specificity of 90.6%. Most cancers were classified with a high level of confidence, with the CNN score close to 1 for NPCs and an area under the curve (AUC) of 0.96, contributing to the early differentiation of benign from malignant nasopharyngeal lesions [20]. Yishu Deng et al. [21] demonstrated in a large cohort that non‐contrast MRI can be used as an alternative to contrast‐enhanced magnetic resonance imaging (ceMRI) to differentiate any T‐phase NPC from benign hyperplasia.

Jaesung Heo et al. [22] collected 12 400 validated endoscopic images and screened out 5576 images related to the tongue, using the DenseNet169 algorithm to improve the early diagnosis rate of tongue cancer.

Hao Xiong et al. [23] constructed and trained a deep convolutional neural network (DCNN)‐based diagnostic system using 13 721 images of laryngeal cancer (LCA), precancerous laryngeal lesions (PRELCA), benign laryngeal tumors (BLT), and normal tissues and found that DCNN has high sensitivity and specificity for the automatic differentiation of LCA and PRELCA from BLT and normal tissues images, which is helpful for the early diagnosis of LCA. Gilbert et al. [24] used 64‐channel multidetector CT technology to acquire images while the patient was holding or blowing and inhaling to evaluate the practical benefit of these targeted images for improving the accuracy of TNM staging in LCA. This technique allows imaging during speech, swallowing, or articulation, providing a new perspective on the staging of LCA.

Sun et al. found that color Doppler ultrasound (CDUS) had better sensitivity and accuracy than MRI in diagnosing cervical lymph node metastases in thyroid cancer. The combination of CDUS and MRI in diagnosing cervical lymph node metastases in thyroid cancer is more accurate. It can provide a more reliable basis for preoperative evaluation and treatment planning [25].

While multimodal digital imaging has markedly enhanced early detection rates for head and neck tumors (HNTs), several challenges hinder its universal adoption. A primary obstacle is the lack of standardized international thresholds for key imaging parameters, such as the apparent diffusion coefficient (ADC) and total lesion glycolysis‐tumor (TLG‐T), which results in significant inter‐institutional variability. Furthermore, while certain advanced techniques—such as MRI integrated with convolutional neural networks (CNNs) for nasopharyngeal carcinoma (NPC) diagnosis—have reached the clinical application stage in endemic regions, others remain restricted. For instance, non‐contrast MRI is still in the small‐scale verification phase due to its limited sensitivity in non‐endemic populations. Additionally, the high cost of PET/CT remains a barrier to its implementation in primary care settings, highlighting an urgent need to improve the clinical readiness of more affordable, AI‐enhanced digital imaging solutions.

#### 3.1.2 Application of Digital Imaging to the Treatment of Head and Neck Tumors

Before treating HNTs, it is necessary to completely segment the area invaded by the tumor and perform preoperative planning. Digital imaging‐based on CT/MRI can help clinicians construct high‐precision 3D anatomical models of HNTs to better show the location of tumors, arteries, and veins [26].

Mui et al. analyzed the role of dynamic contrast enhanced magnetic resonance imaging (DCE‐MRI), diffusion‐weighted magnetic resonance imaging (DW‐MRI), and magnetic resonance spectroscopy (MRS) in the monitoring of NPC treatment. They found that apparent diffusion coefficient (ADC) in the thickened area of the nasopharyngeal wall that remained after treatment was associated with a good treatment response. In addition, ADC correlated with histopathological parameters of HNSCC. MRS enables the non‐invasive detection of metabolites in tumors, facilitating the monitoring of treatment responses. Changes in DCE‐MRI parameters can predict the response to neoadjuvant chemotherapy in patients with NPC. This study suggests that MRI can aid in predicting treatment response and prognosis during NPC treatment monitoring [27]. Wang Zhuo et al. demonstrated that radiomics based on enhanced CT combined with traditional clinical imaging features can intuitively and quantitatively personalize the prediction of induction chemotherapy (ICT) efficacy in patients with locoregionally advanced nasopharyngeal carcinoma (LA‐NPC), which is superior to a single model, and it can be used as a non‐invasive efficacy prediction tool [28].

Li et al.'s [29] three deep learning (DL) models based on MRI images of LCA before and after treatment demonstrated better performance than TNM staging.

While digital imaging shows significant promise for monitoring treatment response, several bottlenecks remain. Most radiomics models are currently derived from single‐center data, which often leads to high overfitting risks and suboptimal performance in external validation. Specifically, integrated models combining DCE‐MRI and radiomics for predicting ICT efficacy are still in the small‐scale clinical verification phase and have yet to be incorporated into formal clinical guidelines. Furthermore, the absence of robust real‐time imaging technology hinders the dynamic adjustment of treatment protocols. The clinical translation of intra‐operative digital imaging is further complicated by technical hurdles, including image distortion and the need for high‐speed, real‐time data transmission.

#### 3.1.3 Application of Digital Imaging to the Prognosis Assessment of Head and Neck Tumors

Sex, age, ethnicity and TNM stage are key clinical factors affecting the prognosis and mortality of HNT patients [30]. Digital imaging can now be used to assess the prognosis of patients with HNTs and predict the survival of patients treated with concurrent chemotherapy [31, 32]. Huang et al. [33] demonstrated that the risk of death increases with the number of parapharyngeal subspaces involved. They suggested using the degree of parapharyngeal space involvement (PPSI) to optimize the homogeneity of T3.

Huang et al. [34] established a CT‐based scale for HNTs to evaluate the resectability of locally advanced thyroid cancer, with an overall accuracy of 78.9%–89.4%. Chen et al. [32] found that patients with high‐risk NPC who received ICT combined with concurrent chemoradiotherapy (CCRT) had better distant metastasis‐free survival (DMFS) at 3 years than those who received CCRT alone (*p* = 0.0340), while patients at low‐risk who received ICT + CCRT had similar DMFS to patients who received CCRT alone.

Chan et al. conducted a prospective study on the prognostic value of imaging parameters in NPC using DCE‐MRI, DWI, and 2‐deoxy‐2‐[fluor‐18]fluoro‐D‐glucose PET (18F‐FDG PET)/CT. They found that the combination of pretreatment DCE‐MRI and 18F‐FDG PET/CT imaging biomarkers could help predict survival in patients with advanced NPC. Integrating MRI perfusion with PET and plasma epstein–barr virus (EBV) information may help clinicians develop the most effective individualized management strategy [35]. Alessi et al. [36] systematically assessed the prognostic value of various 18F‐FDG PET/CT parameters in patients with NPC and found, for the first time, that total lesion glycolysis—tumor (TLG‐T) parameters were closely related to overall survival (OS) and disease‐free survival (DFS) in these patients.

Siow et al. extracted radiological parameters from contrasted T1‐weighted MRI images. They assessed prognostic performance by calculating the incremental area under the curve (IAUC) and found that the radiomics model was validated in the test cohort (IAUC = 0.580). Additionally, MRI radiomics proved valuable in predicting survival outcomes in patients with hypopharyngeal carcinoma treated with CCRT [31]. Ng et al. [37] comprehensively evaluated the prognosis of patients with oropharyngeal or hypopharyngeal squamous cell carcinoma undergoing chemotherapy and radiotherapy using three imaging techniques, that is, DCE‐MRI, DWI, and 18F‐FDG PET/CT. By constructing a prognostic scoring system, they accurately stratified patient survival.

Li et al. studied MRI images of LCA after treatment, which is of great significance for predicting the prognosis of LCA and is helpful for clinical decision‐making [29]. Xie et al.'s [38] T‐staging based on head and neck MRI and N/M staging based on 18F‐FDG PET/CT improved the long‐term prognosis of primary LCA. Zhao et al. [39] used MRI images to predict the number of metastatic lymph nodes in LCA.

Imaging‐based prognostic models can optimize the limitations of TNM staging, but most models do not integrate molecular features (e.g., HPV/EBV status) and clinical treatment plans, leading to low prediction accuracy for heterogeneous tumors. 18F‐FDG PET/CT TLG‐T parameters are in clinical research stage and have not been used as routine prognostic indicators. The clinical readiness of MRI radiomics for hypopharyngeal cancer prognosis is relatively high, but it needs to be further verified by multi‐center large sample studies.

### 3.2 Progress of AI in Head and Neck Tumors

With the continuous advancement of technology, AI utilizes computers to mimic certain human thought processes and intelligent behaviors, thereby enabling computers to achieve higher‐level applications. Among these, machine learning (ML) can conduct simulated learning on computers using vast amounts of data and subsequently make predictions about samples. AI and ML technologies hold the potential to predict treatment outcomes, analyze data, and develop personalized treatment approaches tailored to patients' specific characteristics [40]. Machine learning that is grounded in deep neural network models and methods is known as DL. The most crucial technical characteristic of DL lies in its capacity to automatically extract features. Among them, the most prevalent representative algorithm is the CNN. CNN is a DL approach and a feedforward neural network that incorporates convolutional computations and exhibits a deep‐structured architecture. It possesses the ability of representation learning and can perform translation‐invariant classification of input information in accordance with its hierarchical structure. CNN has been extensively applied to various tasks within computational pathology models, including segmentation, object detection, and image classification. The use of AI/ML‐based models in the management of HNTs is a novel domain [41].

#### 3.2.1 Application of AI in Head and Neck Tumors Diagnosis

AI‐based medical image analysis systems can significantly improve the diagnostic accuracy of clinicians for early HNTs [42]. It often processes and analyzes a large number of images to assist in determining the anatomical positions of the pharynx and larynx [3, 43, 44, 45], and in predicting the benign or malignant nature of tumors [46, 47]. Furthermore, it helps clinicians detect and locate HNTs more quickly and accurately and is expected to achieve automation in diagnosis and treatment evaluation [48].

Hellstrom et al. used CNN to classify head and neck tumors from PET images. The model that performed best for this task was the deep augmentation model, with a median AUC of 85.1%. The four models had the highest sensitivity for tongue root, pyriform sinus, and oral tumors, ranging from 83.3% to 97.7%, 80.2% to 93.3%, and 70.4% to 81.7%, respectively. They also had good sensitivity in detecting follicular thyroid carcinoma, papillary carcinoma, and salivary gland mucinous epidermoid carcinoma (91.7%–100%) [49].

LCA is one of the most common malignant HNTs. CNN is helpful for the early and faster diagnosis of LCA [23, 50]. Zhang et al. [51] used DL and stimulated Raman scattering (SRS) microscopy technology to achieve rapid and accurate intraoperative histological diagnosis of laryngeal squamous cell carcinoma. Xiong et al. [23] analyzed laryngoscope images using a CNN and compared its findings with those of endoscopists with 10–20 years of experience. The sensitivity of the CNN was 0.731, the specificity was 0.922, and the overall accuracy was 0.867, which was comparable to those of experienced physicians.

Qi et al. utilized DL technology based on ultrasound images to assist in the diagnosis of extensive thyroid extrathyroidal extension (ETE) of thyroid cancer. The performance of the DL model in both the internal and external test sets was superior to that of the clinical model, and it was comparable to that of experienced radiologists in terms of diagnostic performance [52]. Assaad et al. [53] developed a DL model using images obtained from high‐resolution scanners and mobile phone cameras to determine the pathological diagnosis of thyroid cancer. Zhu et al. [54] combined AI with frozen sections to facilitate pathologists' identification of thyroid cancer categories. Lee et al.'s [55] DL‐based computer‐aided diagnosis (CAD) system can accurately stage lymph node metastasis (LNM) in the neck of patients with thyroid cancer, with an area under the receiver operating characteristic curve (AUROC) of 0.953.

While AI models have substantially enhanced the early detection rates of head and neck tumors (HNTs), several critical barriers to their widespread adoption remain. A primary challenge is the “black box” nature of these algorithms; a lack of explainability combined with subjective data annotation often leads to low clinical acceptance among practitioners. Furthermore, the developmental stage of these technologies varies by malignancy: deep learning (DL) models for diagnosing extrathyroidal extension (ETE) in thyroid cancer have transitioned into clinical use at tertiary hospitals, whereas convolutional neural network (CNN) models for laryngeal cancer remain in the technical verification phase due to a shortage of standardized laryngoscopic datasets. Additionally, poor interoperability between AI diagnostic systems and diverse imaging hardware continues to hinder large‐scale clinical implementation.

#### 3.2.2 The Application of AI in the Treatment of Head and Neck Tumors

AI is continuously optimizing treatment planning and radiotherapy protocols for HNTs, and providing clinical guidance for adjuvant therapy selection [56]. The integration of AI with digital imaging enables more precise delineation of tumor boundaries and facilitates preoperative segmentation. Although some delineation results still require further refinement, automated segmentation methods have improved work efficiency and enhanced the accuracy of intraoperative tumor resection areas [57, 58, 59, 60, 61, 62, 63, 64]. Meanwhile, when applied to radiotherapy, AI can improve the detection and analysis of lymph nodes [65, 66] and even automate radiation therapy (RT) workflows in the complex domain of HNTs treatment [67].

Kearney et al. [68] applied AI to intensity‐modulated radiotherapy (IMRT) planning for head and neck tumors, and found that deep learning models with a U‐net architecture could predict dose distributions from contour data, which improved the accuracy of dose prediction while reducing the time and workload involved in radiotherapy planning. For salivary gland cancer, AI‐based radiotherapy planning can reduce the radiation dose to the parotid gland by 30% while ensuring tumor coverage, effectively protecting salivary gland function [68, 69]. Howard et al. [56] constructed three (ML) models for data analysis, demonstrating that ML models can assist in clinical decision‐making, guide adjuvant therapies for patients with head and neck tumors, and help reduce the risk of overtreatment. Additionally, Sher et al. [70] revealed that AI‐integrated hybrid instructions contribute to optimally guiding radiotherapy planning for head and neck tumors. In robot‐assisted surgery, AI can reduce intraoperative bleeding and postoperative complications in head and neck tumor surgeries [69]. Wang et al. [71] indicated that cytological slices based on unannotated deep learning are beneficial for research into new targeted therapies for thyroid molecular cancer.

AI optimizes radiotherapy planning and surgical navigation, but the automatic segmentation of tumor boundaries still needs manual correction, and the accuracy of AI models for lymph node detection is low for small lymph nodes (< 5 mm). AI‐based IMRT planning is in clinical application stage in large oncology centers, while AI‐assisted robot surgery is in pilot application stage due to high equipment costs and lack of professional training for surgeons. In addition, there is no unified clinical guideline for AI‐assisted treatment of HNTs, and the responsibility boundary of AI decision‐making is not clear.

#### 3.2.3 Application of AI in Prognosis Evaluation for Head and Neck Tumors

Tumor size, depth of invasion, tumor grade, vascular invasion status, lymph node metastasis, and the size, number, and location of metastases are all important prognostic factors [72]. As patients' expectations for survival increase, improving the quality of life of patients with HNTs, including prolonging survival, reducing pain, and alleviating swallowing problems, is also part of our research on the prognosis of HNT therapies [73, 74]. The application of AI can better detect the disease and improve patient care [75, 76]. Additionally, AI is valuable for predicting the prognosis of patients with HNTs undergoing radiotherapy. Moreover, AI can make more accurate predictions about local recurrence, distant metastasis, and side effects of radiotherapy for HNTs [29, 77, 78, 79, 80].

Zhong et al. [81] developed a DL‐based radiomics model that could predict the prognosis of different treatment regimens based on the pretreatment MRI images of patients with locally advanced NPC and recommend the best treatment plan accordingly. OuYang et al. processed MRI images by selecting a 2.5D CNN to simplify monitoring for NPC recurrence. The AUCs of the AI model in the internal and external validation cohorts were 0.92 and 0.88, respectively, with sensitivities of 79.5% and 74.3% and specificities of 91.0% and 92.8%. The CNN had comparable sensitivity to MRI [82]. Gu et al. developed a DL‐based method for predicting the progression‐free survival (PFS) of patients with NPC. Combining clinical features and DL‐derived radiomics features (DeepMTS‐Score and AutoRadio‐Score) can significantly improve the prediction accuracy of PFS of patients with NPC [83].

Sun et al. explored the relationship between contrast‐enhanced CT (CECT) images and the proliferative state of tongue squamous cell carcinoma (TSCC) by constructing a CNN model. The research results showed that the CNN model could predict the proliferative state of TSCC with 65.38% accuracy and an AUC value of 0.7172 [84].

Valizadeh et al. found that the combination of DL and radiomics has significant potential in detecting LNM of thyroid cancer. The AUC of both the training and internal validation sets exceeded 85%, with high sensitivity and specificity of 85% [85].

Song et al. developed and validated a DL model based on CT, which integrates the characteristics of primary tumors and metastatic cervical lymph nodes (LN) and can significantly classify patients with p16+ oropharyngeal squamous cell carcinoma (OPSCC) into high‐risk and low‐risk groups. The model developed an independent prognostic indicator for DFS and OS. Additionally, it helped to optimize the treatment plan for p16+ OPSCC [86].

AI‐based prognostic models have higher accuracy than traditional clinical models, but most models only predict short‐term prognosis (1–3 years) and lack long‐term follow‐up data. DL radiomics models for NPC prognosis are in the clinical verification stage, and AI models for predicting thyroid cancer lymph node metastasis are in the clinical application stage. The main bottleneck of clinical transformation is the lack of standardized data sharing platforms, leading to the inability of AI models to be effectively verified in different populations.

### 3.3 Progress of 3D Technology in Head and Neck Tumors

#### 3.3.1 Application of 3D Technology to the Diagnosis of Head and Neck Tumors

Gilbert uses 3D technology combined with multi‐row spiral CT and MRI to enable doctors to more accurately assess the extent of tumor invasion. Specialized laryngeal CT scanning techniques, such as while the patient holds their breath or blows air through a straw, can clearly display the movement of the vocal cords, helping to confirm whether the tumor has spread to the vocal cords or other important structures. This scanning method not only aids in staging the tumor but also helps in optimizing plans for surgery and radiotherapy [24]. Additionally, 3D‐printed models can also help patients gain a more intuitive understanding of their condition [87].

**FIGURE 2:**
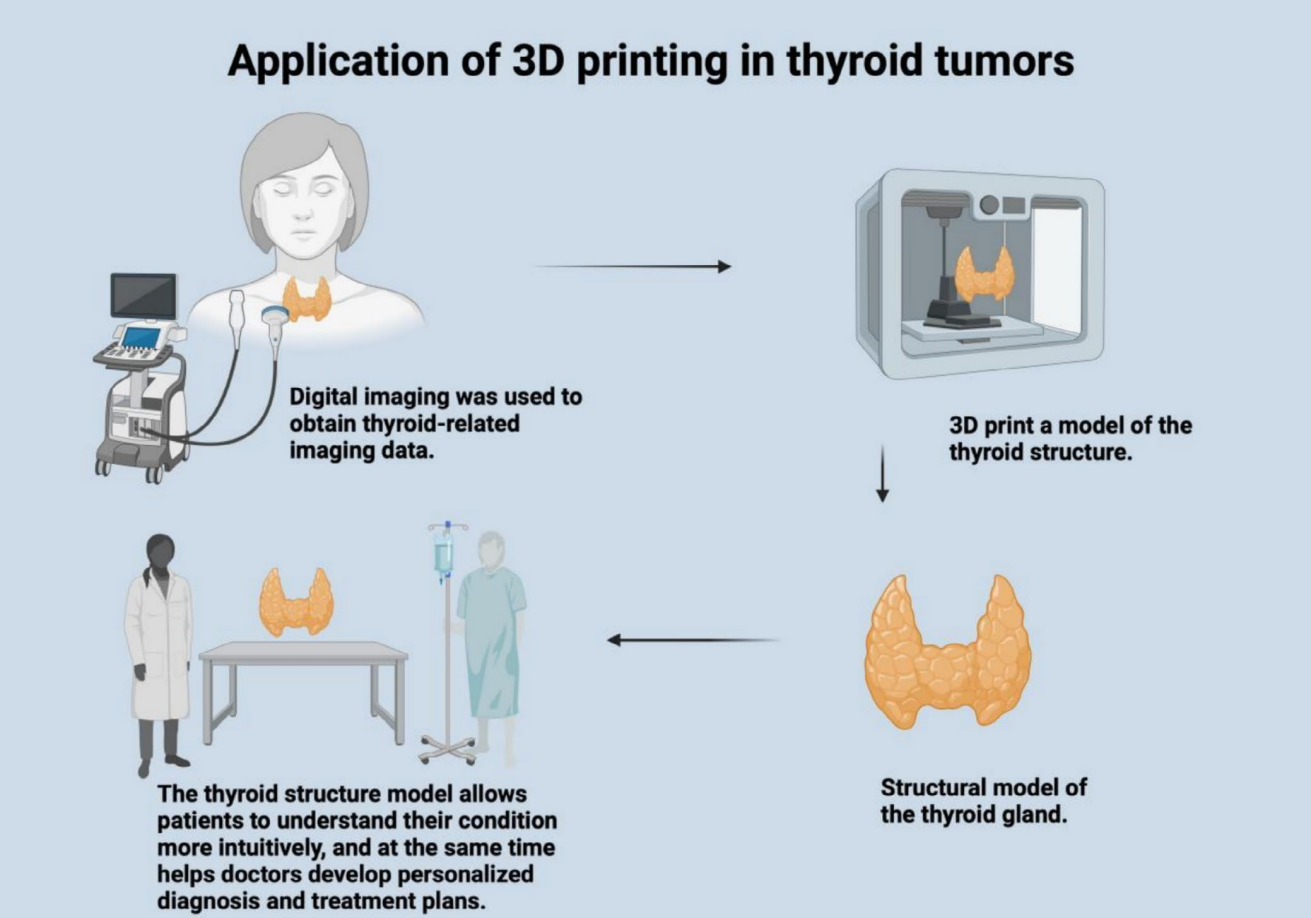
This illustration shows 3D printing's use in thyroid tumors: Digital imaging gets thyroid data, which is 3D—printed into a thyroid structure model. This model helps patients understand their condition and doctors make personalized diagnosis and treatment plans.

3D technology improves the accuracy of tumor invasion assessment, but the resolution of 3D models is limited by the original imaging data, and it is difficult to detect micro‐invasive lesions (< 2 mm). 3D technology combined with multi‐row spiral CT is in clinical application stage for laryngeal cancer staging, while 3D‐printed models for patient communication are in small‐scale promotion stage due to high production costs. In addition, the lack of standardized 3D model reconstruction protocols leads to inconsistent results among different institutions. Additionally, 3D‐printed models can also help patients gain a more intuitive understanding of their condition (Figure 2).

#### 3.3.2 Application of 3D Technology in the Treatment of Head and Neck Tumors

In head and neck surgeries, innovative methods using 3D organoids have been developed to predict squamous cell carcinoma and thyroid regeneration [88].

Ma et al. can generate models of tumors and related anatomical structures using 3D printing technology based on patients' CT or MRI images. These models are used for preoperative planning, helping surgeons determine the optimal resection path and extent, and providing real‐time navigation during the surgery to reduce damage to healthy tissues [89].

Zhu et al. designed and printed 3D personalized surgical guides before the particle implantation surgery for salivary gland cancers based on the patients' CT data. After the surgery, the facial nerve function of all patients returned to normal within 1 year [90]. Hidden lymph node metastasis refers to the presence of cancer cells in lymph nodes that conventional imaging techniques and physical examinations cannot detect.

Wang et al. used the decision fusion model DLRad_DB (combining 3D DL, 2D DL, radiomics, and clinical data) to detect hidden LNM of LSCC, with a high AUC value (0.89–0.90). Their model was significantly superior to clinical models. The model is expected to reduce unnecessary lymph node dissection and prophylactic radiotherapy for patients with cN0 disease [91].

Park et al. compared the diagnostic performance of contrast‐enhanced 3D gradient echo in identifying thyroid cartilage tumor invasion in patients with hypopharyngeal cancer to 2D rotational echo T1 WI and found that the former was superior in diagnosis, providing a more accurate preoperative staging of hypopharyngeal cancer [92]. For hypopharyngeal cancer, 3D‐printed surgical stents can maintain the patency of the upper respiratory tract during surgery and reduce the risk of postoperative airway stenosis [92, 93].

Parikh et al. introduced 3D culture technology (patient derived organoids (PDO) and organotypic culture (OTC)) to study HNSCC. PDO can be generated from multiple tissue samples and cultured long‐term. PDO can capture tumor genetic and expression heterogeneity, predicting responses to various therapies. OTC can simulate the tumor‐stroma interface and be used to study tumor invasion mechanisms and evaluate treatment effects [93].

While 3D printing and 3D organoids offer significant potential for the treatment of head and neck tumors (HNTs), their clinical integration remains uneven. Currently, 3D‐printed surgical guides have reached the clinical application stage for salivary gland and thyroid cancers; however, their use in oral cavity and hypopharyngeal cancers is still largely confined to the research phase. Similarly, 3D organoid technology remains in the preclinical stage, primarily due to the complexities of simulating the tumor microenvironment and the prohibitive costs associated with culture. Furthermore, the biocompatibility of 3D‐printed materials requires further optimization, as the long‐term safety of these applications in clinical settings has yet to be fully established.

#### 3.3.3 Application of 3D Technology to Prognosis Evaluation of Head and Neck Tumors

Tian Hao et al. utilized digitalization combined with 3D printing technology to assist in the resection of lesions, and a personalized titanium mesh was used for the reconstruction of the thyroid cartilage. The repair was performed using adjacent muscle flaps in the larynx and its surroundings, resulting in improved postoperative eating and speech functions, shorter hospital stays, shorter recovery periods, and significant improvement in quality of life [94].

Ziegler et al. used 3D printing technology in a pre‐layered median anterior frontal flap for sub‐total nasal reconstruction. The patient's postoperative nasal shape recovered well, and breathing was normal [95].

3D technology‐based personalized reconstruction significantly improves the quality of life of HNT patients, but the long‐term efficacy (> 5 years) of 3D‐printed titanium mesh for thyroid cartilage reconstruction is still unclear, and the technology is in clinical application stage only in tertiary hospitals. 3D printing for nasal reconstruction is in pilot application stage due to the complex anatomical structure of the nasal cavity and high surgical difficulty. The main limitation of clinical promotion is the high cost of 3D printing and the lack of professional training for reconstructive surgeons. Leading to the inability of AI models to be effectively verified in different populations. A summary of these digital medicine applications is provided in Table 1.

**TABLE 1:** Summary of the application of digital medicine in the diagnosis and treatment of head and neck tumors.

Category	Diagnosis	Treatment	Prognosis
Digital imaging	Tumor segmentation, early diagnosis [19, 20, 23]	Detection of treatment response [27, 28]	Prediction of patients' chemotherapy response [32, 35]
AI	Improved early tumor detection rate, boundary delineation [23, 49, 52]	Optimization of radiotherapy plans, assistance in surgical navigation, reduction of intraoperative bleeding and postoperative complications [56, 68, 69]	Prediction of patients' survival rate and recurrence risk [81, 82, 83]
3D technology	Optimization of preoperative planning [24, 89]	Functional reconstruction, shortening of surgical time [89, 90, 94]	Personalized structure, improvement of patients' quality of life [94, 95]

## 4 Perspectives on Digital Medicine in Head and Neck Tumors

With the development of digital medicine, HNT diagnosis and treatment systems will undergo multi‐dimensional technological innovations. Regarding technological integration and innovation, integrating genomics, imaging, and real‐time monitoring data can be used to build a dynamic treatment decision‐making system. The core goal is to develop lightweight AI tools (such as mobile diagnostic assistants) to significantly enhance the accessibility of primary medical care. Additionally, transfer learning and federated learning technologies at the algorithm level should be adopted to overcome data bottlenecks, and generative AI should be used to simulate tumor heterogeneity and evolution processes, providing new methods for drug resistance research. Regarding breakthroughs in treatment technologies, 3D bioprinting technology will achieve patient‐specific tumor microenvironment modeling. When combined with high‐throughput screening platforms, it can significantly improve the efficiency of targeted drug development. This technology, combined with augmented reality (AR)/virtual reality (VR), can not only optimize the accuracy of surgical navigation but also reshape the clinical physician training system. Furthermore, establishing standardized and ethical frameworks is crucial. It is necessary to build an international standard data sharing platform to promote algorithm transparency and formulate clinical guidelines for AI‐assisted diagnosis, thereby clearly defining responsibility boundaries. Digital medicine is profoundly transforming the traditional HNT diagnosis and treatment model. Combining technological innovation with clinical practice to achieve a full‐chain upgrade from precise diagnosis to personalized prognosis management is urgently required to gradually improve patient survival rates and quality of life.

## Author Contributions

**Bin He:** conceptualization, methodology, investigation, project administration, writing – original draft, writing – review and editing, visualization. **Yanghao Hu:** conceptualization, writing – original draft. **Zhikai Wang:** investigation, visualization. **Yejingwen Tong:** investigation, visualization. **Shengqi Gan:** investigation, visualization. **Yaojie Zhu:** writing – review and editing. **Chongyang Wang:** writing – review and editing. **Dong Ye:** funding acquisition, project administration, supervision, writing – review and editing.

## Funding

This work was supported by grants from Ningbo Top Medical and Health Research Program (2023030514); Ningbo Clinical Research Center for Otolaryngology Head and Neck Disease (2022L005); 2024 Ningbo Public Welfare Science and Technology Plan Key Project (2024S032); Ningbo Natural Science Foundation (2023J213); the key project of the Ningbo Education Science Planning in 2025 (2025YZD001); 2024 Teaching and Research Project of Ningbo University (JYXM2024122); 2022 postgraduate course construction project of Medical School of Ningbo University; Key Project of Huili Foundation (2022ZD003); and Ninghai Science and Technology Project (05).

## Ethics Statement

The authors have nothing to report.

## Consent

The authors have nothing to report.

## Conflicts of Interest

The authors declare no conflicts of interest.

## Data Availability

Data sharing is not applicable to this article as no new data were created or analyzed in this study.
